# Pleural fluid proteomics from patients with pleural infection shows signatures of diverse neutrophilic responses: The Oxford Pleural Infection Endotyping Study (TORPIDS-2)

**DOI:** 10.1183/13993003.00010-2025

**Published:** 2025-07-10

**Authors:** Nikolaos I. Kanellakis, Elie Antoun, Kiki Cano-Gamez, Julia Chu, Nikita Manoharan, Georgina Berridge, Iolanda Vendrell, Zheqing Zhang, John P. Corcoran, Alguili Elsheikh, Tao Dong, Roman Fischer, Justin P. Whalley, Julian C. Knight, Najib M. Rahman

**Affiliations:** 1 Nuffield Department of Medicine, Chinese Academy of Medical Sciences (CAMS) Oxford Institute, University of Oxford, Oxford, UK; 2 Laboratory of Pleural Translational Research, CAMS Oxford Institute, Nuffield Department of Medicine, University of Oxford, Oxford, UK; 3 Oxford Centre for Respiratory Medicine, Churchill Hospital, Oxford University Hospitals NHS Foundation Trust, Oxford, UK; 4 National Institute for Health Research Oxford Biomedical Research Centre, University of Oxford, Oxford, UK; 5 Medical Research Council (MRC) Translational Immune Discovery Unit (MRC TIDU), MRC Weatherall Institute of Molecular Medicine, Radcliffe Department of Medicine (RDM), University of Oxford, Oxford, UK; 6 Centre for Human Genetics, Nuffield Department of Medicine, University of Oxford, Oxford, UK; 7 Discovery Proteomics Facility, Target Discovery Institute, Nuffield Department of Medicine, University of Oxford, Oxford, UK; 8 Center for Cancer Cell Biology, Immunology and Infection, Chicago Medical School, Rosalind Franklin University of Medicine and Science, Chicago, IL, USA; 9 These authors contributed equally to this work

## Abstract

**Background:**

Pleural infection is a complex disease with poor clinical outcomes and increasing incidence worldwide, yet its biological endotypes remain unknown.

**Methods:**

We analysed 80 pleural fluid samples from the PILOT study, a prospective study on pleural infection, using unlabelled mass spectrometry. A total of 449 proteins were retained after filtering. Unsupervised hierarchical clustering and Uniform Manifold Approximation and Projection analyses were used to cluster samples and pathway analysis was performed to identify the biological processes. Protein signatures as identified by the pathway analysis were compared to microbiology as defined by 16S rRNA next-generation sequencing. Spearman and exact Fischer's methods were used for correlation assessment.

**Results:**

Higher neutrophil degranulation was correlated with increased glycolysis (odds ratio (OR) 281, p<2.2×10^−16^) and pentose phosphate activation (OR 371.45, p<2.2×10^−16^). Samples dominated by *Streptococcus pneumoniae* exhibited higher neutrophil degranulation (OR 12.08, p=0.005), glycolysis (OR 11.4, p=0.006) and pentose phosphate activity (OR 12.82, p=0.004). Samples dominated by anaerobes and Gram-negative bacteria exhibited lower neutrophil degranulation (OR 0.15, p=0.01), glycolysis (OR 0.14, p=0.01) and pentose phosphate activity (OR 0.07, p=0.001). Increased activity of the liver and retinoid X receptors pathway was associated with lower risk of 1-year mortality (OR 0.24, p=0.04).

**Conclusions:**

These findings suggest that pleural infection patients exhibit diverse responses of neutrophil-mediated immunity, glycolysis and pentose phosphate activation, which are associated with microbiology. Therapeutic targeting of the liver and retinoid X receptors pathway with agonists is a possible treatment approach.

## Shareable abstract

**Pleural infection patients present with diverse endotypes of the disease** https://bit.ly/41LTull

## Graphical abstract

**Figure:**
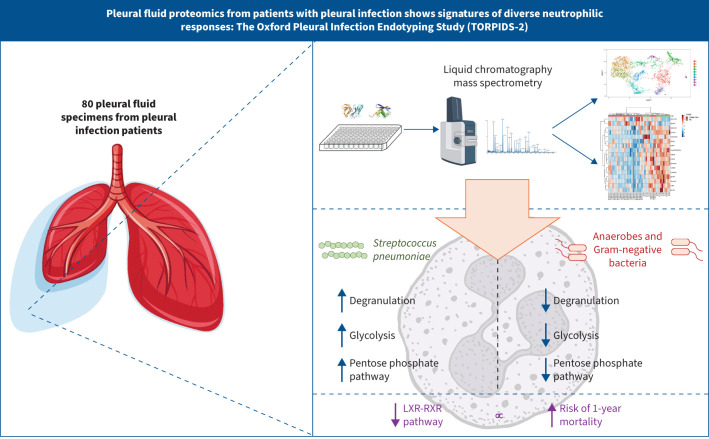
Overview of the study. LXR-RXR: liver and retinoid X receptor.

## Introduction

Pleural infection is a severe disease with increasing incidence worldwide and is characterised by substantial associated morbidity and mortality [1]. Although it is accepted that the disease is heterogeneous, and there is a validated clinical prediction score (RAPID) [2, 3], the biological endotypes of pleural infection remain elusive and pleural fluid-specific criteria to assess the intrapleural response are not available. A better understanding of pleural infection subtypes could lead to improved treatment strategies and clinical outcomes.

Clinical guidelines for pleural infection patients recommend hospital admission, pleural fluid drainage and administration of antibiotics. However, the processes of recovery, progression and eventual clinical outcome differ significantly [1]. A subgroup of patients exhibits ineffective or failed intrapleural fibrinolysis leading to the development of fibrous septations, which further complicates treatment. The degradation of plasminogen by neutrophil elastase and the increased protein levels of plasminogen activator inhibitor 1 (PAI-1) have been implicated to contribute to the fibrinolytic deficiency; however, the exact molecular basis remains elusive [4]. Approximately 30% of patients do not respond to initial treatment and require invasive treatments, including surgical drainage or intrapleural enzyme therapy [5].

Our study (The Oxford Pleural Infection Endotyping Study, TORPIDS-2) applied mass spectrometry to pleural fluid specimens (n=80) from a prospective clinical study which collected samples, clinical data and the adjudicated clinical outcomes (PILOT) [3]. Our primary aims were to investigate if endotypes of pleural infection exist, characterise variation in intrapleural immune responses and investigate the association between endotypes and high-precision bacterial patterns. The associations between endotypes and clinically important outcomes (1-year survival and need for surgery) were analysed, as well as the association between pleural fluid plasminogen and neutrophil elastase.

## Material and methods

### Study design, samples and ethics

TORPIDS-2 is a prospective follow-up of the PILOT and TORPIDS studies [3, 6]. In TORPIDS-2, 80 pleural fluid specimens, deeply clinically phenotyped [3] and accompanied by high-fidelity bacteriology data, were subjected to unlabelled mass spectrometry.

### Sample collection for the PILOT study

Samples were collected prospectively at enrolment for the PILOT study [3]. Patients were recruited on identical clinical and laboratory criteria between 1 May 2013 and 1 January 2017. Evidence of infection was assessed by the recruiting physician on the basis of fever, elevated peripheral white blood cell count or elevated serum inflammatory markers (C-reactive protein). Detailed inclusion and exclusion criteria are described in the supplementary methods.

Ethical and regulatory approval for the study was obtained from Medical Sciences Interdivisional Research Ethics Committee (IDREC, R74885/RE001). The trial is registered with ClinicalTrials.gov (NCT06513689).

### Sample selection for the TORPIDS-2 study

Pleural fluid specimens (n=80) were randomly selected to represent eight different microbiological patterns (tables 1 and 2) as identified in the TORPIDS [6] study using high-fidelity 16S rRNA next-generation sequencing [6]. In a given sample, a specific pathogen or group of pathogens was defined as dominant when their (next-generation sequencing) reads were at least 98% of the total reads. Aliquots of previously non-thawed acellular pleural fluid were selected for analysis to ensure protein integrity.

**TABLE 1:** Cohort demographics for the 80 patients whose specimens were used in the TORPIDS-2 study

Characteristic	
**Participants**	80 (100)
**Age (years)**	60.5±17.9
**Sex**	
Female	26 (33)
Male	54 (67)
**1-year survival**	
Yes	65 (81)
No	15 (19)
**Hospital stay (days)**	15.2±12.7
**Surgery**	
Yes	7 (9)
No	73 (91)
**RAPID score^#^**	
Low	36 (46)
Middle	31 (39)
High	12 (15)

Data are presented as mean±sd or n (%). ^#^: n=79 for RAPID score because one sample was excluded from the results.

**TABLE 2:** Microbiology of the samples

Dominant bacteria	Samples (n)	Age (years), mean±sd
***Streptococcus pneunomiae***	10	48.4±30.6
***Staphylococcus aureus***	8	62.9±26.4
***Streptococcus anginosus* group**	10	64.6±12.7
***Enterobacteriaceae***	9	62.9±13.9
**Anaerobes**	10	57.9±18.6
**Mixed anaerobes and Gram-negative**	14	52.6±25.6
**Gram-negative**	10	59.0±19.8
**Gram-positive**	9	65.9±24.2

The table presents the microbiology for the samples that were used for the study. Each sample belongs to only one group. The microbiology was determined by 16S rRNA next-generation sequencing in a previous study [6].

### Liquid chromatography mass spectrometry

An Evosep One (liquid chromatography) with timsTOF Pro mass spectrometer (Bruker, Billerica, MA, USA) high-throughput platform was used for the sample analysis. A detailed description can be found in the supplementary methods.

### Quality control and analysis of mass spectrometry data

Samples with fewer than 125 proteins were excluded from the analysis as previously described [7, 8]. The proteomic datasets were filtered to retain only those proteins detected in at least 50% of the sample injections, resulting in the retention of 449 proteins for subsequent analyses, as with previous research [7, 8].

Following the selection of proteins, data normalisation of the raw intensity values and bias correction were performed using the variance stabilisation normalisation method [9]. To address the issue of missing values, a common challenge in high-throughput omics datasets, an imputation strategy based on k-nearest neighbours was used. The k-nearest neighbours imputation method leverages the similarity between samples to predict missing values, assuming that samples with similar overall profiles are likely to have similar values for missing data points.

### Unsupervised hierarchical clustering

Unsupervised hierarchical clustering analyses were performed using the Euclidean distance and the complete-linkage method, with the Pheatmap R package used for graph plotting. Additionally, Uniform Manifold Approximation and Projection (UMAP) analysis was performed. These were performed on the normalised protein values.

### Differential protein analyses

Differential protein analyses between groups were performed calculating the fold change for each protein. Statistical significance was determined using the Mann–Whitney U test. The Benjamini–Hochberg (non-negative) false discovery rate was used to adjust the p-values for multiple comparisons, with family-wise error rate=0.05.

### Pathway analysis

Pathway analysis was performed using Ingenuity Pathway Analysis. p-values were calculated using the right-tailed Fischer's exact test and the Benjamini–Hochberg false discovery rate method was used to correct for multiple testing.

### Correlation analyses

Spearman's correlation coefficient was used to assess the correlation between sets of relative protein expression. Fisher's exact test was used for protein abundance compared to discrete clinical measurements. 1-year survival was treated as a binary variable.

### Statistical significance

p-values <0.05 were considered statistically significant. The analysis was performed with R (version 3, www.r-project.org).

### Data availability

Mass spectrometry data generated in this study have been deposited in the PRoteomics IDEntifications Database (PRIDE) (dataset identifier: PXD054108).

## Results

### High-throughput proteomics identified different protein signatures in pleural fluid specimens from pleural infection patients

A total of 80 acellular pleural fluid specimens from patients with confirmed community-acquired pleural infection (table 1) were prospectively collected upon recruitment for the PILOT study [3] and subjected to unlabelled mass spectrometry analysis. The samples were selected to represent eight different patterns of bacterial microbiology as identified by 16S rRNA next-generation sequencing in our previous study [6] (table 2 and supplementary methods). Overall, we identified 892 proteins. One sample did not pass quality control and thus was excluded from the analysis (see Methods) [7]. The dataset was further filtered to keep only the proteins detected in at least half of the samples, resulting in the retention of 449 proteins (supplementary file 1).

**FIGURE 1:**
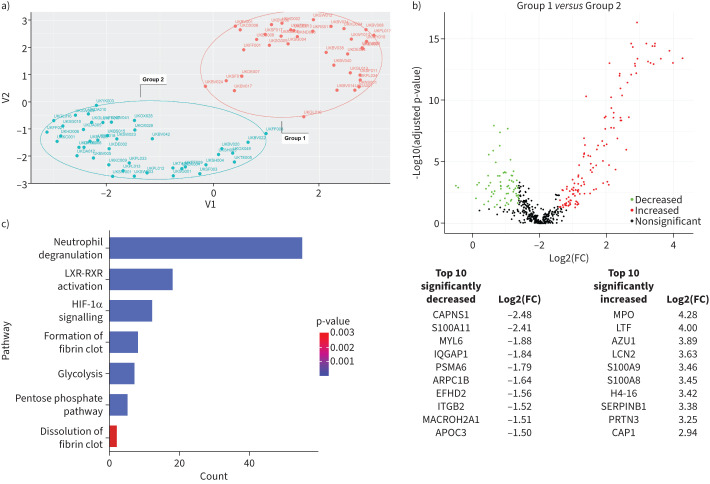
Pleural fluid samples derived from pleural infection patients showed diverse proteomic expression. a) Uniform Manifold Approximation and Projection (UMAP) plot for the whole proteome after protein filtration and normalisation. Each dot represents a sample. The samples were clustered into two separate and independent cohorts (Group 1 and Group 2). b) Differential protein expression analysis. Volcano plot presenting the differential protein expression analysis for Group 1 compared with Group 2 samples as separated in the UMAP above. Green dots represent proteins significantly decreased in Group 1 compared to Group 2; red dots represent proteins significantly increased in Group 1 compared to Group 2; black dots represent proteins that are below the fold-change (FC) threshold and nonsignificant. Statistical significance was determined using the Mann–Whitney U test. c) Significant pathways as identified by the pathway analysis of the significant proteins as identified in b. The colour represents the p-value and the count the number of identified proteins.

Unsupervised hierarchical clustering for the filtered dataset revealed different patterns of protein expression (supplementary figure S1) among the samples. UMAP separated the samples in two non-overlapping and independent clusters of protein expression, denoted from here on as Group 1 and Group 2 (figure 1a).

To better understand the endotypes, we sought to investigate the biological differences between these two clusters. Differential protein expression analysis comparing Group 1 to Group 2 revealed 184 proteins (119 increased, 65 decreased) to be statistically significantly differentially expressed (adjusted p-value <0.05) with an absolute fold change ≥2 (figure 1b and supplementary file 2). Eight of the 10 most increased proteins in Group 1 are known to be involved in neutrophil-mediated immunity, seven are serine proteases, and H4 histone 16 (H4-16) is a component of neutrophil extracellular traps (NETs) (figure 1b) [10]. Of the significantly decreased proteins, calpain small subunit 1 (CAPNS1) stabilises the proteases calpain-1 and calpain-2 [11]. CAPNS1 knockout mice with acute peritonitis exhibit lower neutrophil recruitment, a reduced capacity to clear bacteria and an increased risk of progressing to bacteraemia [12]. Proteasome 20S subunit α6 (PSMA6) is part of the 20S core proteasome complex; suppression of PSMA6 *via* small interfering RNA reduces the activation of NF-κB [13]. Apolipoprotein C3 (APOC3) triggers the NLR family pyrin domain containing 3 (NLRP3) inflammasome in human monocytes [14]. Integrin subunit β2 (ITGB2) plays a role in neutrophil migration and *ITGB2* gene mutations cause leukocyte adhesion deficiency 1, in which neutrophils fail to migrate from the circulation to the tissues [15].

To discover the underlying biological processes associated with the differentially expressed proteins, pathway analysis was performed. The pathways for neutrophil degranulation, glycolysis, pentose phosphate, hypoxia inducible factor 1α (HIF-1a) signalling activation and liver and retinoid X receptor (LXR-RXR) activation were significantly different between the groups (figure 1c).

### Pleural infection patients exhibited different levels of neutrophil degranulation activity, which was associated with the underlying microbiology

Neutrophil degranulation (p=2×10^−57^) was the top identified pathway. Unsupervised hierarchical clustering for the identified proteins of this pathway (figure 2a) separated the samples into two main groups revealing different patterns of neutrophil activity. UMAP analysis for the same proteins clustered the samples into two distinct and non-overlapping groups (figure 2b). The first cluster was dominated by Group 1 samples (35 out of 38, 92%) and the other by Group 2 samples (39 out of 41, 95%; odds ratio (OR) 188.09, p=3.00×10^−16^). Group 1 samples showed higher levels of neutrophil degranulation compared to Group 2 (figure 2a). Group 1 samples with high neutrophil activity were more likely to exhibit higher white blood count, for which they belonged to the fourth quartile (supplementary figure S2; OR 4.12, p=0.02).

**FIGURE 2:**
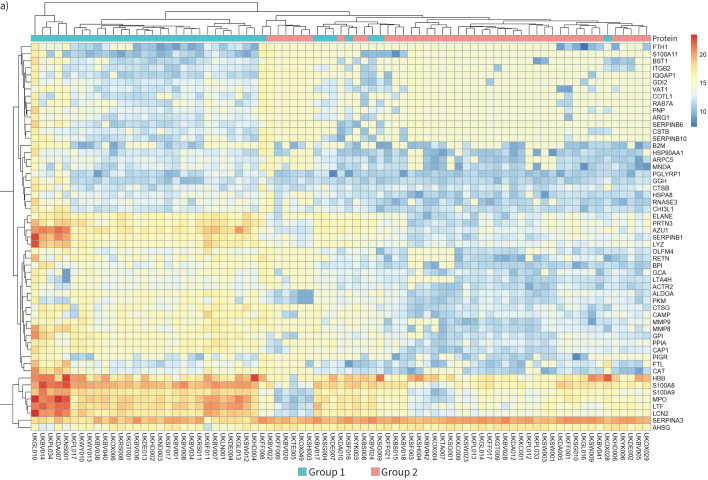
Pleural infection patients exhibited different levels of neutrophil degranulation activity. a) Heatmap of unsupervised hierarchical clustering for the identified proteins of the neutrophil degranulation pathway. Each row is a protein of the pathway and each column a sample. The colours of the cells represent the relative protein abundance and the colours at the top represent the groups as defined in figure 1. b–e) Uniform Manifold Approximation and Projection plots for the proteins of the neutrophil degranulation pathway. Each dot is a sample, and the colour represents the groups as defined in figure 1 (b), dominance of *Streptococcus pneumoniae* (c), dominance of *Streptococcus anginosus* (d) and dominance of a mixture of strict anaerobes and Gram-negative bacteria (e).

**FIGURE 2:**
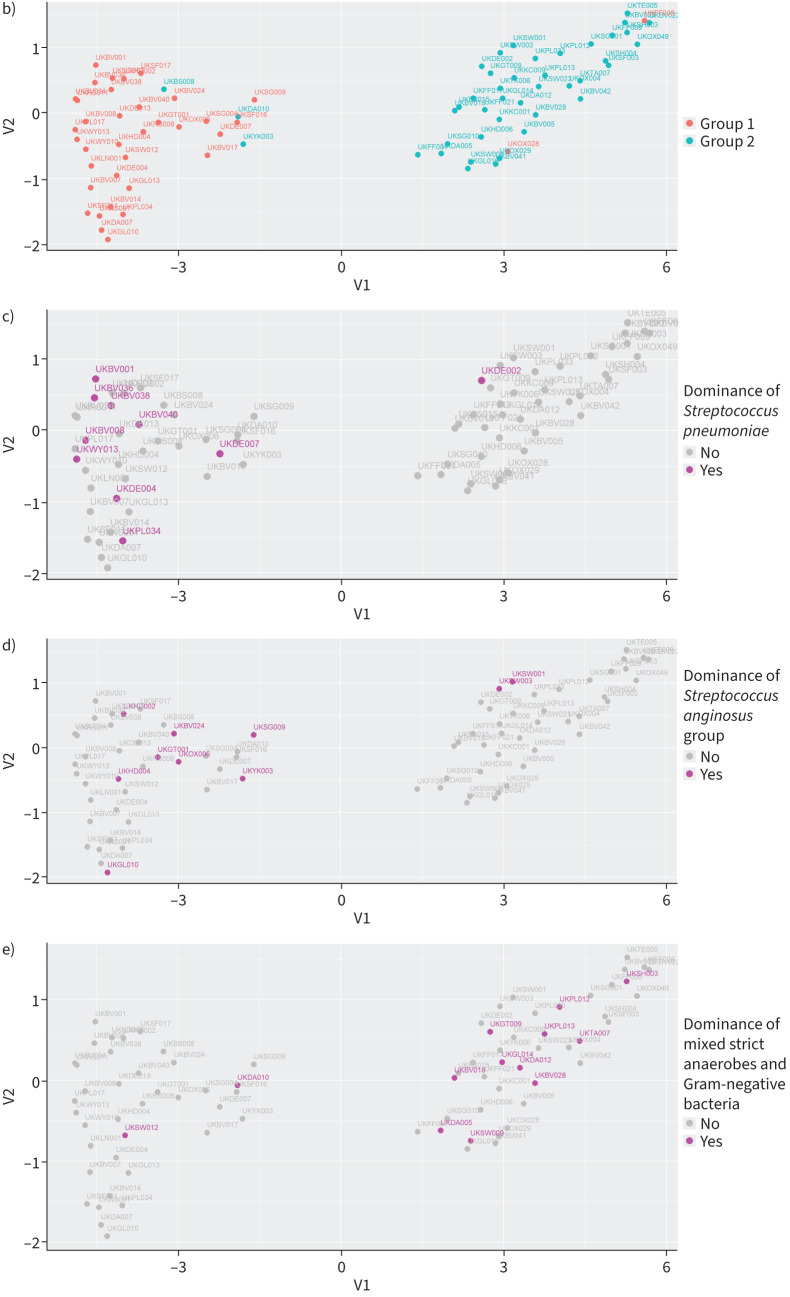
Continued.

Next, we investigated the association between neutrophil activity and microbiological patterns. Samples dominated by *Streptococcus pneumoniae* (figure 2c; OR 12.08, p=0.005) or *S. anginosus* (OR 5.10, p=0.04) were associated with high neutrophil degranulation (figure 2c, d). By contrast, samples showing a mixture of strict anaerobes and Gram-negative bacteria were associated with the lower neutrophil degranulation cluster (figure 2e; OR 0.15, p=0.01). No associations were identified for samples dominated by *Enterobacteriaceae*, *Staphylococcus aureus*, strict anaerobes or Gram-negative or Gram-positive bacteria. However, when samples dominated by a mixture of strict anaerobes and Gram-negative bacteria were pooled together with samples dominated by Gram-negative and samples dominated by strict anaerobes, they were clustered in the lower neutrophil degranulation activity cohort (OR 0.22, p=0.002).

### Samples with increased neutrophil degranulation exhibited higher metabolic activity

**FIGURE 3:**
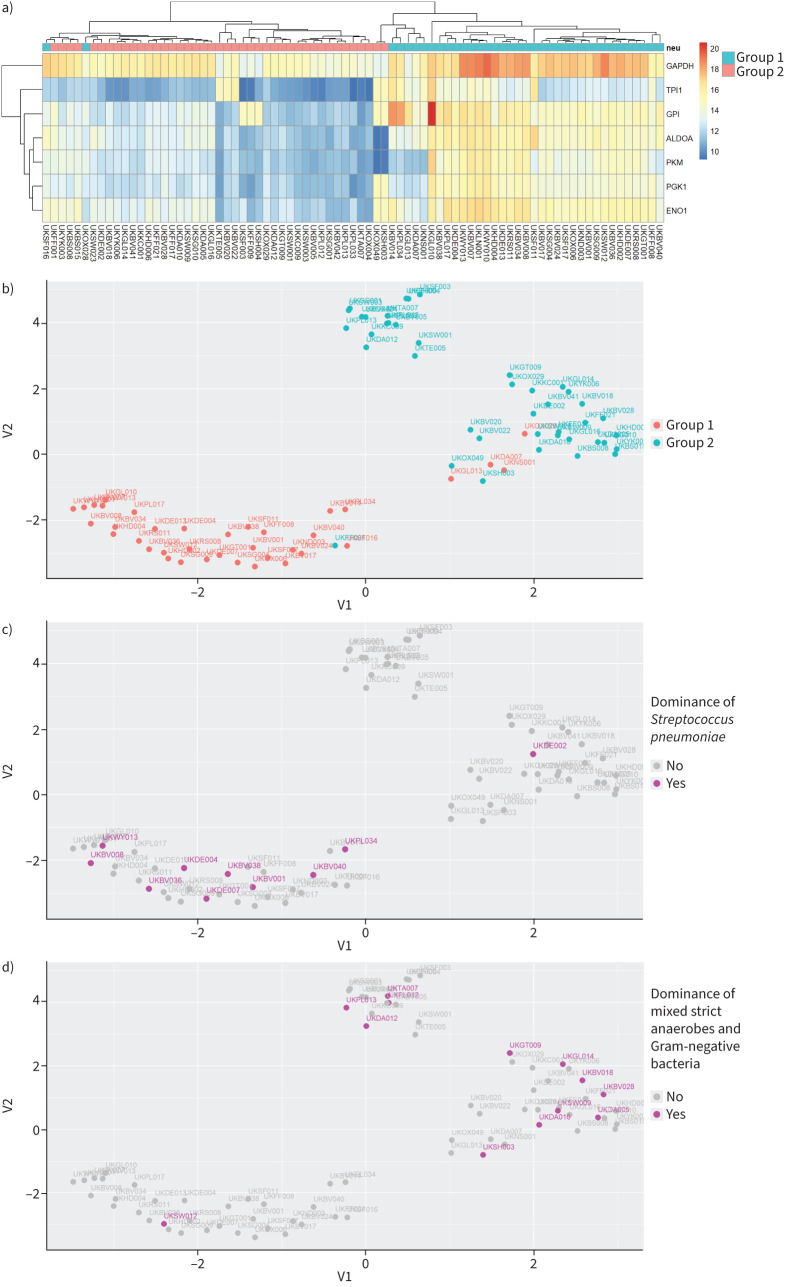
Pleural infection patients showed different levels of glycolytic metabolism. a) Heatmap of unsupervised hierarchical clustering for the identified proteins of the glycolysis pathway. Each row is a protein of the pathway and each column a sample. The colours of the cells represent the relative protein abundance and the colours at the top the groups as defined in figure 1. b–d) Uniform Manifold Approximation and Projection plot for the identified proteins of the glycolysis pathway. Each dot is a sample, and the colours represent the groups as defined in figure 1 (b), dominance of *Streptococcus pneumoniae* (c) and dominance of a mixture of strict anaerobes and Gram-negative bacteria (d).

The pathways of glycolysis and pentose phosphate are essential for cellular metabolism. Unsupervised hierarchical clustering and UMAP analysis for the glycolysis pathway (p=2×10^−10^) segregated the samples into two distinct cohorts (figure 3a, b). One of the clusters was dominated by Group 2 samples (41 out of 42, 98%) and the other by Group 1 samples (33 out of 37, 89%). There was a positive association between neutrophil degranulation activity and glycolysis (OR 281, p<2.2×10^−16^). Group 1 samples with higher neutrophil activity showed increased glycolytic activity compared to Group 2 samples with lower neutrophil activity (figure 3a). Samples dominated by *S. pneumoniae* exhibited higher glycolysis (figure 3c; OR 11.4, p=0.006) while samples with a mixture of strict anaerobes and Gram-negative bacteria showed lower glycolytic activity (figure 3d; OR 0.14, p=0.01).

**FIGURE 4:**
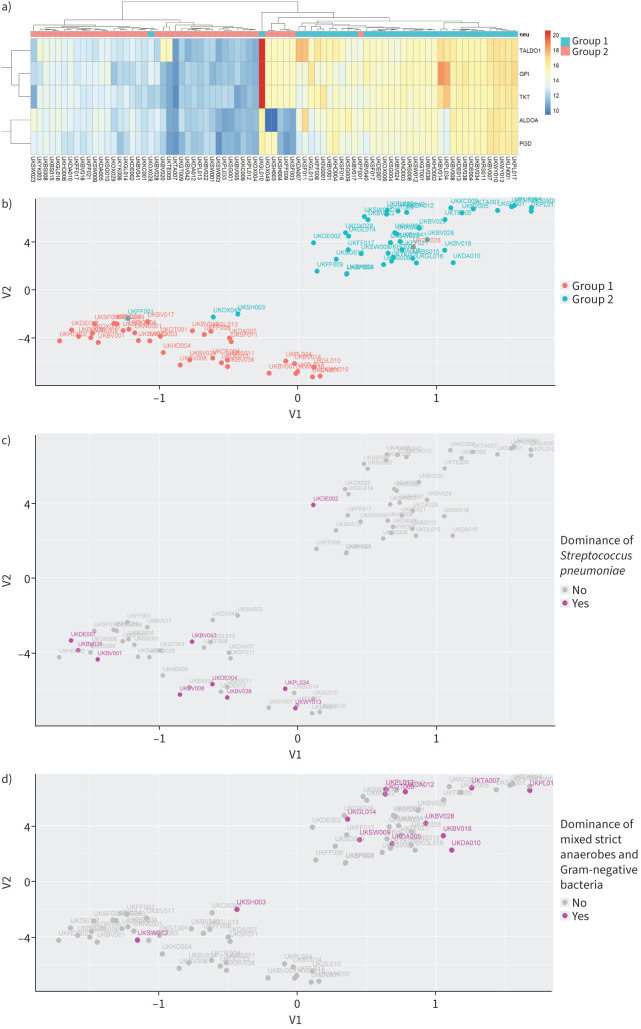
Pleural infection patients displayed different levels of pentose phosphate metabolism. a) Heatmap of unsupervised hierarchical clustering for the identified proteins of the pentose phosphate pathway. Each row is a protein of the pathway and each column a sample. The colours of each cell represent the relative protein abundance and the colours at the top the groups as defined in figure 1. b–d) Uniform Manifold Approximation and Projection plot for the identified proteins of the pentose phosphate pathway. Each dot is a sample. The colours represent the groups as defined in figure 1 (b), dominance of *Streptococcus pneumoniae* (c) and dominance of a mixture of strict anaerobes and Gram-negative bacteria (d).

Unsupervised hierarchical clustering and UMAP analysis for the pentose phosphate pathway (p=7.4×10^−6^) separated the samples into two distinct and non-overlapping cohorts (figure 4a, b). One of the clusters was dominated by Group 1 samples (36 out of 39, 92%) and the other by Group 2 samples (39 out of 40, 98%). As per the glycolytic activity analysis, there was a positive association between pentose phosphate clusters and neutrophil degranulation activity (OR 371.45, p<2.2×10^−16^). Group 1 samples with higher neutrophil activity showed increased pentose phosphate activity compared to Group 2 samples with lower neutrophil activity (figure 4a). Samples dominated by *S. pneumoniae* were more likely to cluster in the high pentose phosphate activity cohort (figure 4c; OR 12.82, p=0.004). Samples dominated by a mixture of strict anaerobes and Gram-negative bacteria clustered in the lower pentose phosphate activity cluster (figure 4d; OR 0.07, p=0.001).

### Samples with increased neutrophil degranulation showed higher HIF-1α signalling activity

HIF-1α signalling is induced under hypoxic conditions. Unsupervised hierarchical clustering revealed different patterns of HIF-1α signalling activation (p=5.13×10^−6^) among the samples (supplementary figure S3). Group 1 samples with higher neutrophil activity showed increased HIF-1α activity compared to Group 2 samples. UMAP analysis spread the samples, ranging from Group 1 (bottom left) to Group 2 samples (top right). Despite the subgroups being homogeneous, there was no distinct separation between the two cohorts (supplementary figure S4).

### Higher activation of the LXR-RXR pathway was positively correlated with better survival

**FIGURE 5:**
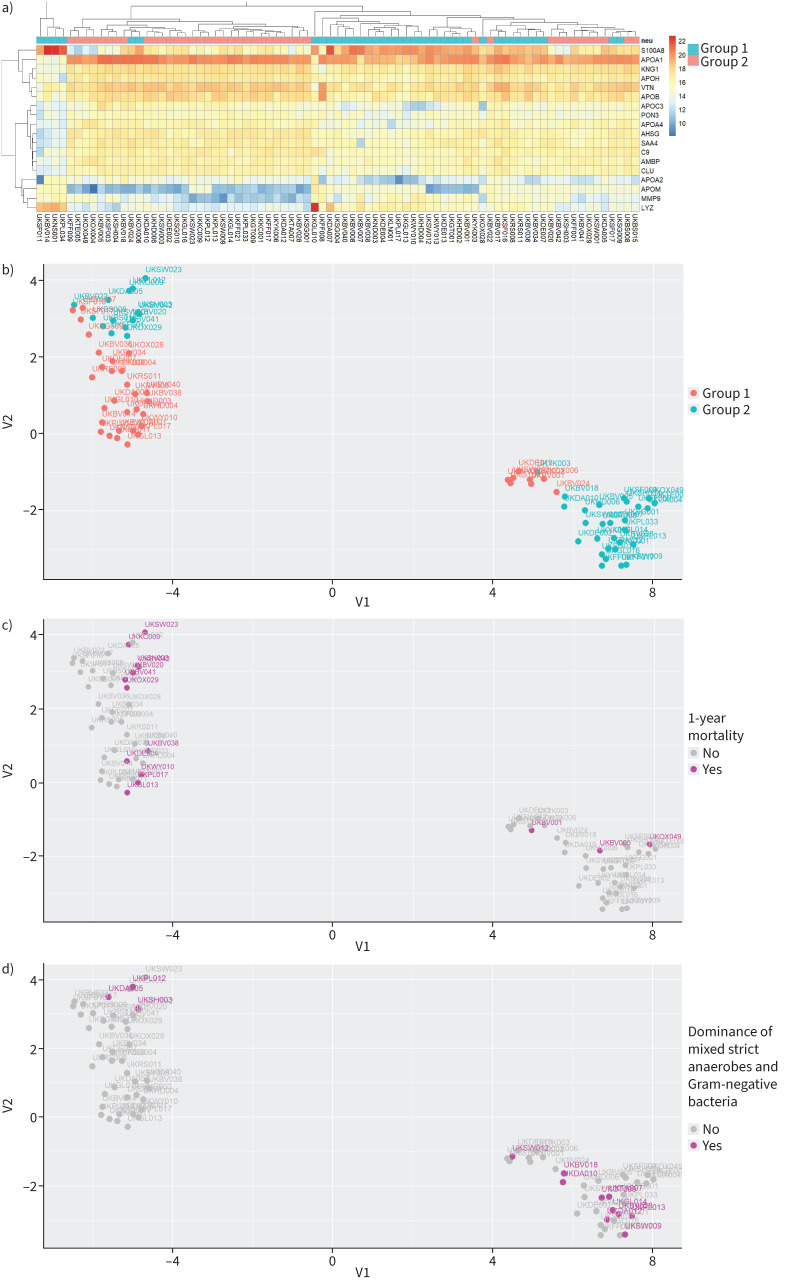
Pleural infection patients exhibited various levels of the liver and retinoid X receptor (LXR-RXR) activation pathway. a) Heatmap of unsupervised hierarchical clustering for the identified proteins of the LXR-RXR activation pathway. Each row is a protein of the pathway and each column a sample. The colours of the cells represent the relative abundance of the proteins and the colours at the top the groups as defined in figure 1. b–d) Uniform Manifold Approximation and Projection plot for the identified proteins of the LXR-RXR activation pathway. Each dot is a sample. The colours represent the groups as defined in figure 1 (b), 1-year survival (c) and dominance of a mixture of strict anaerobes and Gram-negative bacteria (d).

The LXR-RXRs are involved in innate antimicrobial responses, lipid metabolism and cholesterol transport to the liver [16, 17]. Unsupervised hierarchical clustering and UMAP analysis for the LXR-RXR activation pathway (p=1×10^−10^) clustered the samples into two distinct cohorts (figure 5a, b). The first cluster was dominated by Group 1 samples (29 out of 43, 67%) and the other by Group 2 samples (28 out of 36, 77%; OR 7.04, p=9.44×10^−5^). The Group 2 dominant cohort showed higher levels of LXR-RXR activation (figure 5a) and 1-year survival was better in the higher LXR-RXR activity cluster (figure 5c; OR 0.24, p=0.04). Samples dominated by a mixture of strict anaerobes and Gram-negative bacteria were clustered in the Group 2 dominant cohort (figure 5d; OR 5.02, p=0.01).

### The protein levels of intrapleural neutrophil elastase were negatively correlated with plasminogen

The pathway analysis identified the processes of formation of fibrin clot (p=7.94×10^−11^) and dissolution of fibrin clot (p=0.003). Previous studies have suggested that plasminogen deficiency due to degradation by neutrophil elastase is the leading cause of fibrinolysis arrest [4, 18]. In our cohort, plasminogen protein levels exhibited higher expression levels, greater variation and wider distribution compared to PAI-1 (supplementary figures S5 and S6, and supplementary table S1). A strong negative correlation was detected between plasminogen and neutrophil elastase (Spearman's rank rho= −0.66, p<2.2×10^−16^; supplementary figure S7).

## Discussion

This is the first study to apply label-free mass spectrometry to discover and thoroughly explore the intrapleural biological immune response in pleural infection and to correlate protein expression with clinical outcomes. Our data suggest that pleural infection patients exhibit different levels of intrapleural neutrophil activity, and these levels are associated with bacterial cause, neutrophil metabolic activity, HIF-1α activation and the LXR-RXR activation pathway.

Unsupervised hierarchical clustering and UMAP analysis for the whole dataset subdivided the samples into two independent and non-overlapping cohorts. The distinct clustering indicates that pleural infection exhibits diverse pleural endotypes. Several previous studies on infection, including sepsis and COVID-19, have revealed the existence of such different patient subpopulations [7, 19]. A previous study applied mass spectrometry to discover biomarkers that distinguished between patients with pneumonia and complicated or uncomplicated parapneumonic effusions. Bactericidal permeability-increasing protein (BPI), neutrophil gelatinase-associated lipocalin (NGAL), azurocidin 1 (AZU1) and calprotectin (S100A8, S100A9) were validated to have elevated expression in patients with complicated parapneumonic effusions, with BPI showing the highest diagnostic value [20]. In our study, we found that these proteins showed significantly increased expression in the high neutrophil activity group.

For the proteomic data analysis, we followed an unbiased data-driven approach. To this end, we sought to delineate the differences between the two groups, identified in an unsupervised manner. Pathway analysis for the statistically significantly differentially expressed proteins between the two groups identified neutrophil degranulation activity as the most important process. A previous study using a microfluidic infection-on-a-chip device reported that different bacterial species mount diverse neutrophil responses [21]. Consistent with previous findings, our data suggest that different bacterial patterns elicit distinct neutrophil responses, with samples dominated by *S. pneumoniae* showing higher levels of neutrophil activity. Previous studies have shown that pneumonia caused by *S. pneumoniae* triggers an intense inflammatory response [22]. Pneumococcal pneumolysin has been identified as a major virulence factor and key driver of the host inflammatory response [23, 24]. A better understanding of the complex interactions between bacteria and host could elucidate strategies bacteria use to evade immune surveillance and how the immune response itself could cause tissue damage and lead to immunopathology. Knowledge of these mechanisms has the potential to alter patient clinical management through the development of targeted treatments. This will move treatment away from broad-spectrum antibiotics, which is critical in the era of antimicrobial resistance.

Glycolysis is the major means of energy production for neutrophils; however, upon activation they switch to the pentose phosphate pathway to power the oxidative burst [25, 26]. A previous study has shown that *in vitro* stimulation of human neutrophils under normoxic and hypoxic conditions induces the metabolic pathways of glycolysis and pentose phosphate [27]. Our study supports these findings, because the level of neutrophil activity was associated with metabolic activity. Samples which exhibited high neutrophil degranulation displayed a more active metabolism, as indicated by increased activity of the glycolysis and pentose phosphate pathways, compared to the low neutrophil activity samples. Our findings confirm the metabolic plasticity of neutrophils in pleural infection.

In low oxygen environments such as the pleural cavity, neutrophils upregulate HIF-1α though mitochondrial reactive oxygen species, which enables their survival [28–30]. Our data revealed different levels of HIF-1α pathway activation between high and low neutrophil activity samples. It has been shown that hypoxia induces neutrophil degranulation, and this may lead to tissue damage [31, 32]. Our study supports these *in vitro* findings, with high neutrophil degranulation samples showing higher activation levels of the HIF-1α signalling pathway compared to those with low neutrophil degranulation. Consistent with previous studies, our data suggest that HIF-1α activation stimulates protein production of myeloperoxidase, neutrophil elastase, lactotransferrin and matrix metalloproteinase 9 [31, 32].

Our findings indicate that patients with pleural infection who have low activity of the LXR-RXR pathway have shorter survival. No significant correlation was found between survival and the other proteomic signatures. It has been reported that stimulation of the LXR-RXR pathway prevents bacterial-induced macrophage apoptosis [17]. Resident macrophages of the pleural cavity play a crucial role in patrolling the intrapleural environment and promoting the recruitment of immune cells during bacterial infection [33]. Activation of the LXR-RXR pathway induces lipid metabolism and reverse cholesterol transport [34, 35]. Lipid metabolism activity during infection is reduced and patients with sepsis who present with hypocholesterolaemia exhibit increased mortality [36]. Together these findings suggest the potential use of synthetic LXR-RXR agonists like T1317 or GW3965 to boost innate immunity and lipid metabolism in patients with pleural infection who are at increased risk of mortality [17, 37]. Further studies are required to validate this hypothesis.

Suppression of the intrapleural fibrinolytic pathway is a complication of pleural infection and it has been postulated that PAI-1 is responsible for halting fibrinolysis. However, recent studies have described the inflammatory degradation of plasminogen by neutrophil elastase as the principal cause [4, 18]. We identified a strong negative correlation between neutrophil elastase and plasminogen protein levels. The MIST-2 study [5] showed that intrapleural administration of tissue plasminogen activator (tPA) and DNase improved fluid drainage and shortened hospital stay. The authors hypothesised that DNase cleaved extracellular DNA, which increased the viscosity of the fluid. Considering the new findings presented in this study, one possible mechanism of action of DNase in association with tPA may be that it acts to cleave NETs, which contain neutrophil elastase and thus aid fibrinolysis. Patients at risk of intrapleural fibrinolytic deficiency might benefit from intrapleural administration of NETosis inhibitors like protein arginine deiminase 4 inhibitors (JBI-589, GSK484) [38, 39]. More work is needed to validate this hypothesis.

The strengths of our study include its objective design and unbiased protein discovery and data-driven analysis strategy, as well as a large cohort of clinically and microbiologically well-characterised human samples. All specimens were prospectively collected upon hospitalisation under the same inclusion criteria and, to the best of our knowledge, this is the first study applying high-throughput mass spectrometry to phenotype and characterise the intrapleural immune response of pleural infection.

This study has several limitations. Mass spectrometry cannot absolutely quantify protein expression. Moreover, the inter-individual high dynamic range of protein concentration may limit the depth of protein discovery. Some post-translational modifications (*i.e.* phosphorylation, glycosylation) that could affect protein functionality may be hard to detect. For example, PAI-1 is a highly metastable protein, where the concentrations of the total and active protein might differ [40]. Mass spectrometry detects the relative level of a protein in a given sample without the ability to differentiate the levels based on the cell of origin. Furthermore, the size of the study sample did not provide the power to perform thorough comparisons with clinical parameters and clinical outcomes. All pleural fluid specimens were collected from adult patients in the UK.

In conclusion, proteomic analysis of pleural infection specimens revealed different endotypes of the disease and a diverse intrapleural response. Patients exhibited diverse levels of intrapleural neutrophil activation and metabolism, which were associated with different bacterial patterns. Early signals suggest mortality may differ according to baseline proteomic expression.

## Shareable PDF

10.1183/13993003.00010-2025.Shareable1 This PDF extract can be shared freely online.
Shareable PDF

ERJ-00010-2025.Shareable

## Data Availability

This study did not generate new unique reagents. Pleural fluid samples may be available with a completed materials transfer agreement. Mass spectrometry data generated in this study have been deposited in the PRoteomics IDEntifications Database – PRIDE, dataset identifier: PXD054108. This paper does not report original code. Any additional information required to reanalyse the data reported in this paper is available from the lead contact upon request.

## Supplementary material

**Please note:** supplementary material is not edited by the Editorial Office, and is uploaded as it has been supplied by the author. Supplementary material: methods, tables and figures ERJ-00010-2025.Supplement Supplementary file 1 ERJ-00010-2025.File_1 Supplementary file 2 ERJ-00010-2025.File_2
