# Supplementary material for: Pleural fluid proteomics from patients with pleural infection shows signatures of diverse neutrophilic responses: The Oxford Pleural Infection Endotyping Study (TORPIDS-2)
Source: Eur Respir J. 2025 Jul 10;66(1):2500010. doi: 10.1183/13993003.00010-2025 (PMC12256804; doi:10.1183/13993003.00010-2025)
Supplement: Supplementary file 1 [file ERJ-00010-2025.Shareable.pdf]

EUROPEAN RESPIRATORY JOURNAL
ORIGINAL RESEARCH ARTICLE
N.I. KANELLAKIS ET AL.

SHAREABLE PDF

Pleural fluid proteomics from patients with pleural infection shows signatures of diverse neutrophilic responses: The Oxford Pleural Infection Endotyping Study (TORPIDS-2)

Nikolaos I. Kanellakis, Elie Antoun, Kiki Cano-Gamez, Julia Chu, Nikita Manoharan, Georgina Berridge, Iolanda Vendrell, Zheqing Zhang, John P. Corcoran, Alguili Elsheikh, Tao Dong, Roman Fischer, Justin P. Whalley, Julian C. Knight and Najib M. Rahman

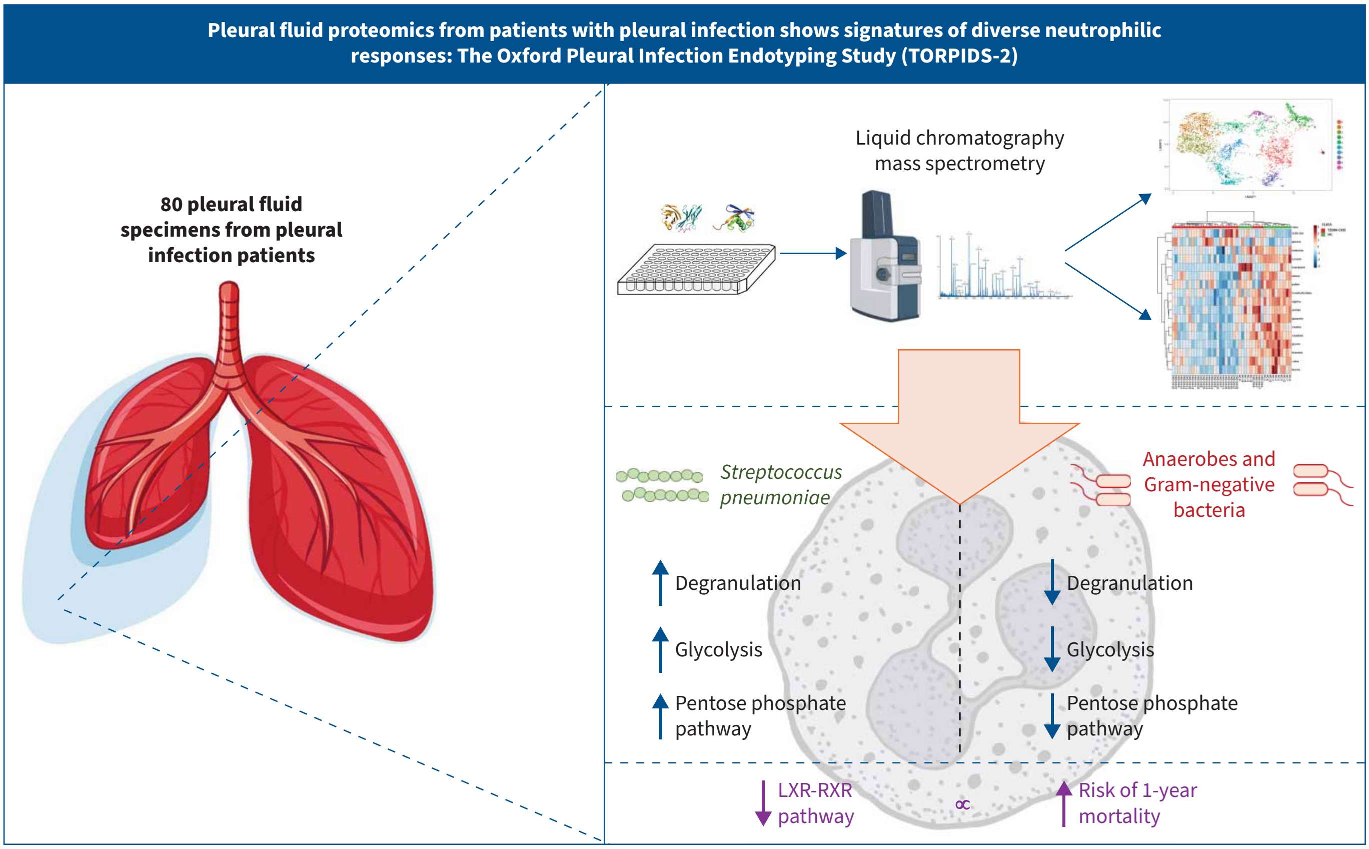

GRAPHICAL ABSTRACT Overview of the study. LXR-RXR: liver and retinoid X receptor.

EUROPEAN RESPIRATORY JOURNAL
ORIGINAL RESEARCH ARTICLE
N.I. KANELLAKIS ET AL.

SHAREABLE PDF

Pleural fluid proteomics from patients with pleural infection shows signatures of diverse neutrophilic responses: The Oxford Pleural Infection Endotyping Study (TORPIDS-2)

Nikolaos I. Kanellakis[1,2,3,4], Elie Antoun[1,5], Kiki Cano-Gamez[6], Julia Chu[1,2], Nikita Manoharan[1,2], Georgina Berridge[7], Iolanda Vendrell[1,7], Zheqing Zhang[1,2], John P. Corcoran[3], Alguili Elsheikh[3], Tao Dong[1,5], Roman Fischer[1,7], Justin P. Whalley[8,9], Julian C. Knight[1,6,9] and Najib M. Rahman[1,3,4,9]

[1]Nuffield Department of Medicine, Chinese Academy of Medical Sciences (CAMS) Oxford Institute, University of Oxford, Oxford, UK. [2]Laboratory of Pleural Translational Research, CAMS Oxford Institute, Nuffield Department of Medicine, University of Oxford, Oxford, UK. [3]Oxford Centre for Respiratory Medicine, Churchill Hospital, Oxford University Hospitals NHS Foundation Trust, Oxford, UK. [4]National Institute for Health Research Oxford Biomedical Research Centre, University of Oxford, Oxford, UK. [5]Medical Research Council (MRC) Translational Immune Discovery Unit (MRC TIDU), MRC Weatherall Institute of Molecular Medicine, Radcliffe Department of Medicine (RDM), University of Oxford, Oxford, UK. [6]Centre for Human Genetics, Nuffield Department of Medicine, University of Oxford, Oxford, UK. [7]Discovery Proteomics Facility, Target Discovery Institute, Nuffield Department of Medicine, University of Oxford, Oxford, UK. [8]Center for Cancer Cell Biology, Immunology and Infection, Chicago Medical School, Rosalind Franklin University of Medicine and Science, Chicago, IL, USA. [9]These authors contributed equally to this work.

Corresponding author: Nikolaos I. Kanellakis (nikolaos.kanellakis@ndm.ox.ac.uk)

Check for updates

Shareable abstract (@ERSpublications)
Pleural infection patients present with diverse endotypes of the disease https://bit.ly/41LTull

Cite this article as: Kanellakis NI, Antoun E, Cano-Gamez K, *et al.* Pleural fluid proteomics from patients with pleural infection shows signatures of diverse neutrophilic responses: The Oxford Pleural Infection Endotyping Study (TORPIDS-2). *Eur Respir J* 2025; 66: 2500010 [DOI: 10.1183/13993003.00010-2025].

This PDF extract can be shared freely online.

Copyright ©The authors 2025.

This version is distributed under the terms of the Creative Commons Attribution Licence 4.0.

This article has an editorial commentary: https://doi.org/10.1183/13993003.00861-2025

Received: 3 Jan 2025
Accepted: 8 March 2025

Abstract

Background Pleural infection is a complex disease with poor clinical outcomes and increasing incidence worldwide, yet its biological endotypes remain unknown.

Methods We analysed 80 pleural fluid samples from the PILOT study, a prospective study on pleural infection, using unlabelled mass spectrometry. A total of 449 proteins were retained after filtering. Unsupervised hierarchical clustering and Uniform Manifold Approximation and Projection analyses were used to cluster samples and pathway analysis was performed to identify the biological processes. Protein signatures as identified by the pathway analysis were compared to microbiology as defined by 16S rRNA next-generation sequencing. Spearman and exact Fischer's methods were used for correlation assessment.

Results Higher neutrophil degranulation was correlated with increased glycolysis (odds ratio (OR) 281, $p<2.2\times10^{-16}$) and pentose phosphate activation (OR 371.45, $p<2.2\times10^{-16}$). Samples dominated by *Streptococcus pneumoniae* exhibited higher neutrophil degranulation (OR 12.08, p=0.005), glycolysis (OR 11.4, p=0.006) and pentose phosphate activity (OR 12.82, p=0.004). Samples dominated by anaerobes and Gram-negative bacteria exhibited lower neutrophil degranulation (OR 0.15, p=0.01), glycolysis (OR 0.14, p=0.01) and pentose phosphate activity (OR 0.07, p=0.001). Increased activity of the liver and retinoid X receptors pathway was associated with lower risk of 1-year mortality (OR 0.24, p=0.04).

Conclusions These findings suggest that pleural infection patients exhibit diverse responses of neutrophil-mediated immunity, glycolysis and pentose phosphate activation, which are associated with microbiology. Therapeutic targeting of the liver and retinoid X receptors pathway with agonists is a possible treatment approach.
