# Supplementary material for: Pleural fluid proteomics from patients with pleural infection shows signatures of diverse neutrophilic responses: The Oxford Pleural Infection Endotyping Study (TORPIDS-2)
Source: Eur Respir J. 2025 Jul 10;66(1):2500010. doi: 10.1183/13993003.00010-2025 (PMC12256804; doi:10.1183/13993003.00010-2025)
Supplement: Supplementary file 2 [file ERJ-00010-2025.Supplement.pdf]

Supplementary Material

Pleural fluid proteomics from patients with pleural infection shows signatures of diverse neutrophilic responses: The Oxford Pleural Infection Endotyping Study (TORPIDS-2)

Nikolaos I. Kanellakis[1,2,3,4,9], Elie Antoun[1,5], Kiki Cano-Gamez[6], Julia Chu[1,2], Nikita Manoharan[1,2], Georgina Berridge[7], Iolanda Vendrell[1,7], Zheqing Zhang[1,2], John P. Corcoran[3], Alguili Elsheikh[3], Tao Dong[1,5], Roman Fischer PhD[1,7], Justin P. Whalley[8,*], Julian C. Knight[1,6,*], and Najib M. Rahman[1,3,4,*]

Affiliations

1. Chinese Academy of Medical Sciences Oxford Institute, Nuffield Department of Medicine, University of Oxford, Oxford, UK
2. Laboratory of Pleural Translational Research, CAMS Oxford Institute, Nuffield Department of Medicine, University of Oxford, Oxford, OX3 9BN, UK
3. Oxford Centre for Respiratory Medicine, Churchill Hospital, Oxford University Hospitals NHS Foundation Trust, Oxford, UK
4. National Institute for Health Research Oxford Biomedical Research Centre, University of Oxford, Oxford, UK
5. Medical Research Council (MRC) Translational Immune Discovery Unit (MRC TIDU), MRC Weatherall Institute of Molecular Medicine, Radcliffe Department of Medicine (RDM), University of Oxford, Oxford, UK
6. Centre for Human Genetics, Nuffield Department of Medicine, University of Oxford, Oxford, UK,
7. Discovery Proteomics Facility, Target Discovery Institute, Nuffield Department of Medicine, University of Oxford, Oxford, UK
8. Center for Cancer Cell Biology, Immunology and Infection, Chicago Medical School, Rosalind Franklin University of Medicine and Science, Chicago, IL, USA
9. Lead contact

Corresponding author:
Nikolaos I. Kanellakis PhD
Laboratory of Pleural Translational Research, CAMS Oxford Institute, Nuffield Department of Medicine, University of Oxford, Oxford, OX3 9BN, UK
Email: nikolaos.kanellakis@ndm.ox.ac.uk

Contents

Supplementary Methods 3

Supplementary Tables... 6

Supplementary Figures 7

Supplementary Methods

Liquid Chromatography and Mass Spectrometry

2 µL of pleural fluid samples were diluted in 200uL PBS and then digested with trypsin (1:10). The resulting peptides were desalted using standard SOLA HRP SPE plates (60309-001, Thermo Fisher Scientific, Waltham, MA), dried overnight in a vacuum concentrator then resuspended into 50uL 0.1% formic acid. An equivalent of 200ng/sample were loaded onto Evo-tips (Evosep Biosystems, Denmark) and analysed by liquid chromatography mass spectrometry (LC-MS/MS) using the Evosep One (LC) - TimsTOF Pro mass spectrometer (Bruker, Billerica, MA) high-throughput platform. Peptides were separated over 21-min gradient (60 sample per day -spd- standard method) using a 150 µm × 8 cm C18 column, bead size 1.5 µm (PepSep, EV-1109, Bruker, Billerica, MA). Mass spectrometry data were acquired in a data-independent mode with parallel accumulation and serial fragmentation (DIA-PASEF). The ion mobility range was set to 0.85 – 1.3 Vs/cm^2. TIMS ion accumulation and ramp times were set to 100 ms and mass spectra were recorded from 100–1700 m/z, with 8 diaPASEF scans per TIMS-MS scan, giving a duty cycle of 0.96 s. Raw files were analysed in DIA-NN[1] version 1.8.1 using an in-silico spectral library generated by DIA-NN with default settings (1 missed cleavage, N-terminal methionine excision was allowed) using a Uniprot human FASTA file containing 20,383 reviewed sequences. MS1 (precursor scan) and MS2 (fragment scan) accuracies were set to 15 ppm, all other settings were left as default.

TORPIDS Study

The TORPIDS study[2] investigated the microbiology of pleural infection and its association with clinically important outcomes (1-year survival, need for surgery). The microbiology of 243 pleural fluid specimens prospectively collected for the PILOT study was assessed with 16S rRNA next generation sequencing.

RAPID Score

The RAPID score is a validated risk prediction score to assess three-month mortality at presentation in adult patients with pleural infection.[3, 4] The RAPID score is using urea (serum), age (years), purulence (yes/no), infection source (community, hospital), and serum albumin. Patients are stratified into three risk groups: low, medium, and high.

Outcomes for the TORPIDS-2 study

The primary outcomes were the demonstration of biologically distinct pleural infection endotypes and intrapleural immune responses and their association with microbial patterns and one-year survival. Secondary outcomes were the association between pleural infection endotypes and need for surgical treatment and the association between levels of pleural fluid plasminogen and neutrophil elastase.

Inclusion and exclusion criteria for the "Prospective validation of the RAPID clinical risk prediction score in adult patients with pleural infection: the PILOT study" clinical trial[3]

Patients were included if they had a clinical presentation consistent with pleural infection as stated in the Methods section of the main manuscript and any of the following criteria:

1. Pleural fluid that was macroscopically purulent, OR
2. Pleural fluid that was positive on culture for bacterial infection, OR
3. Pleural fluid that demonstrated bacteria on Gram staining, OR
4. Pleural fluid with pH ≤7.2 (measured in a blood gas analyser) or low glucose level (≤3mmol/L

1. or ≤55mg/dL) in a patient with clinical evidence of infection, OR

5. Contrast-enhanced CT evidence of pleural infection (consolidation of underlying lung with enhancing pleural collection) in a patient with clinical evidence of infection, alongside exclusion of other sources of infection.

Exclusion criteria for the PILOT study:

1. Age less than 18 years, OR
2. No pleural fluid available for analysis, OR
3. Patient previous pneumonectomy on the side of pleural infection, OR
4. Expected survival less than three months due to co-morbid disease, as judged by the recruiting physician.

Sample and data collection for the "Prospective validation of the RAPID clinical risk prediction score in adult patients with pleural infection: the PILOT study" clinical trial[3]

Pleural fluid and blood specimens were collected at enrolment. The samples were centrifuged at 400g for 15 minutes at room temperature. Supernatants were transferred into sterile 2.0 ml cryovial microcentrifuge tubes and stored in -80 ^{o}C freezers.

Data collected for each study participant included age, smoking status, dental hygiene, comorbidities, chest radiographic findings, dates of hospital admission and discharge, treatment, culture microbiology findings, haemoglobin, C-reactive protein (CRP), full blood count, pleural fluid LDH, and pleural fluid protein. Survival data for each patient was collected from the trial database and cross-validated with the UK national registry. Patients with missing data were excluded from the analysis.

Supplementary Table

Supplementary Table S1

	**PLG**	**PAI-1**
**Mean (SD)**	15.56 (1.08)	11.74 (0.94)
**Median (IQR)**	11.84 (1.60)	11.65 (0.69)
**Variance**	1.16	0.89
**Range**	12.17 – 17.01	10.35 – 16.39

Supplementary Table 3. Statistics for Plasminogen and Plasminogen Activator Inhibitor 1. The table presents the mean, median, variance, and range for the protein levels of Plasminogen and Plasminogen Activator Inhibitor.

Supplementary Figures
Supplementary Figure S1

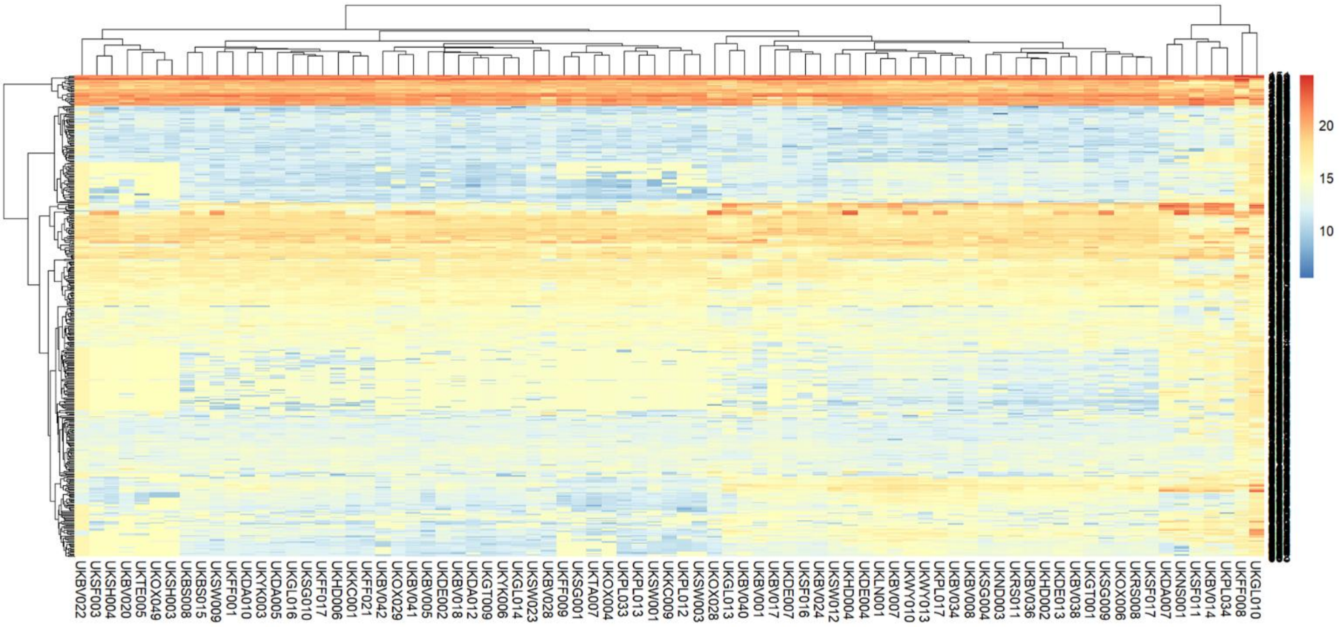

Supplementary Figure S1. The samples present different patterns of protein expression. The figure shows a heatmap of unsupervised hierarchical clustering for the filtered dataset. The algorithm separated the samples into distinct clusters revealing different patterns protein expression within the cohort of the study. Each column is a sample and each row a protein. Cell colours represent the relative abundance of each protein for each of the samples.

Supplementary Figure S2

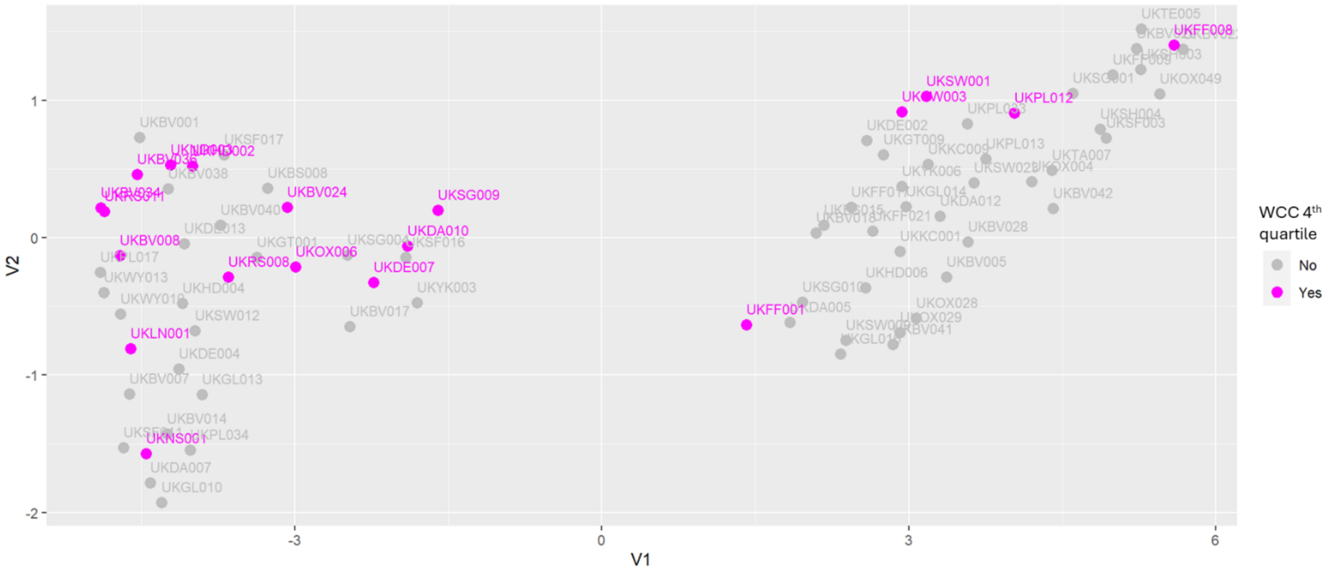

Supplementary Figure S2. Higher neutrophil activation was associated with increased white blood cell count. The figure presents UMAP analysis for the pathway of neutrophil degranulation (Figure 2B). The cohort on the left shows higher neutrophil degranulation activity and was associated with increased white blood cell count (WCC). Each dot represents a sample and the colour the quartile of WCC.

Supplementary Figure S3

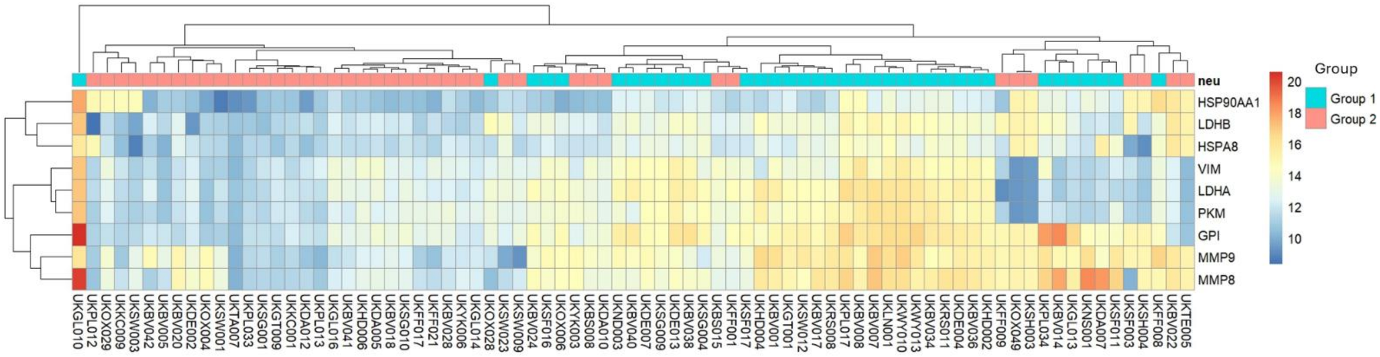

Supplementary Figure S3. Hypoxia Inducible Factor 1a (HIF-1a) signalling pathway. The figure presents a heatmap of unsupervised hierarchical clustering for the pathway of HIF-1a signalling. The algorithm separated the samples into different clusters. Each column is a sample and each row a protein which belongs to the pathway. The colours of the cells represent the relative abundance of each protein for each sample.

Supplementary Figure S4

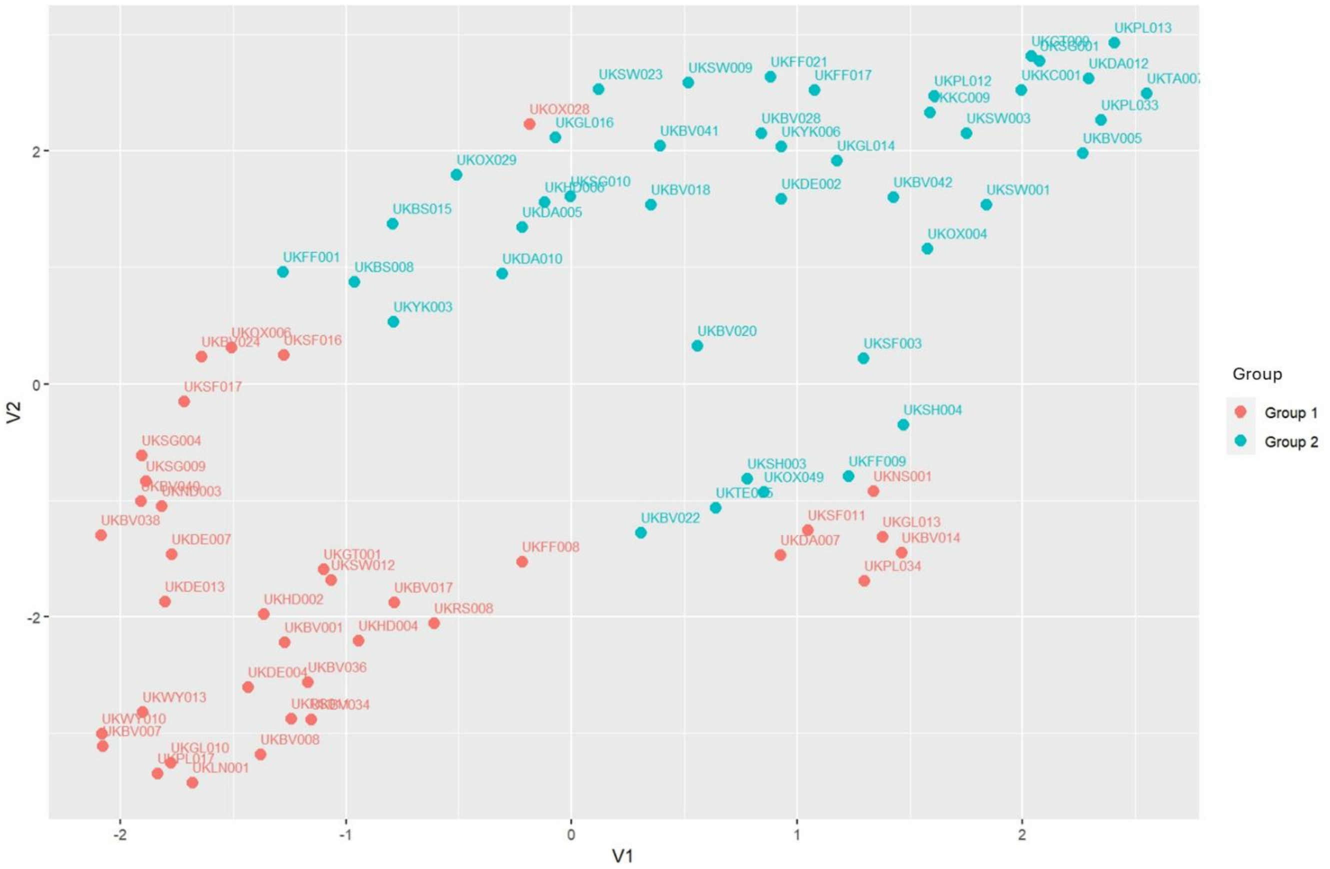

Supplementary Figure S4. Hypoxia Inducible Factor 1a (HIF-1a) signalling pathway. UMAP for the pathway of Hypoxia Inducible Factor 1a (HIF-1a) signalling. The samples were spread into two subgroups. Each dot represents a sample and the colour the group as this was defined in Figure 1.

Supplementary Figure S5

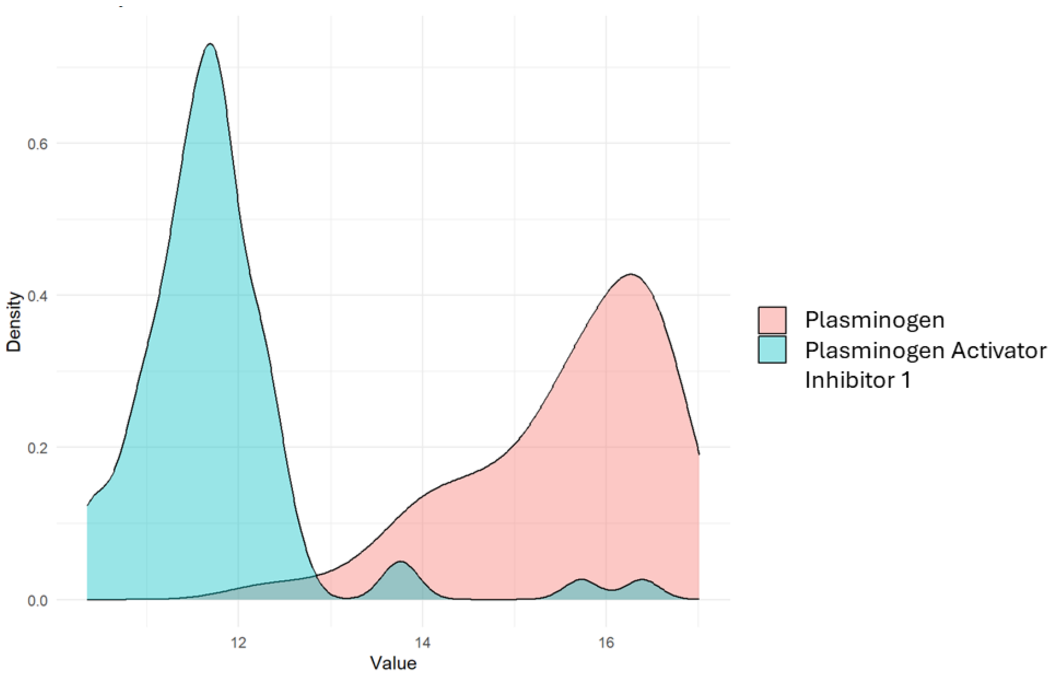

Supplementary Figure S5. Density plots for Plasminogen and Plasminogen Activator Inhibitor 1 proteins levels. Plasminogen showed higher expression levels and greater variation compared to Plasminogen Activator Inhibitor 1.

Supplementary Figure S6

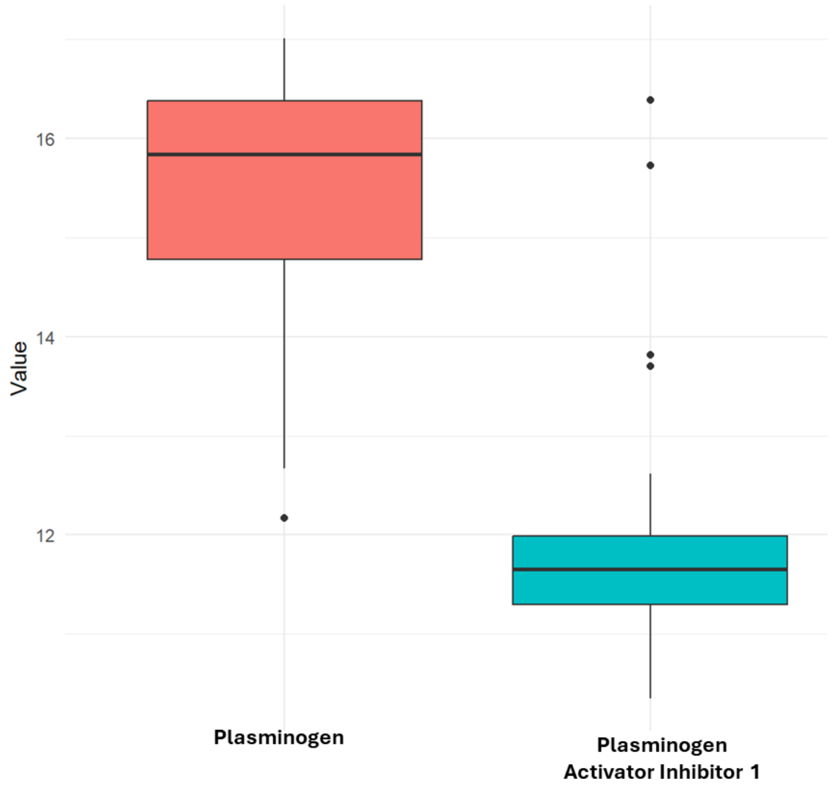

Supplementary Figure S6. Box plots for Plasminogen and Plasminogen Activator Inhibitor 1 protein levels. Plasminogen exhibited higher expression, greater variation, and wider distribution compared to Plasminogen Activator Inhibitor 1.

Supplementary Figure S7

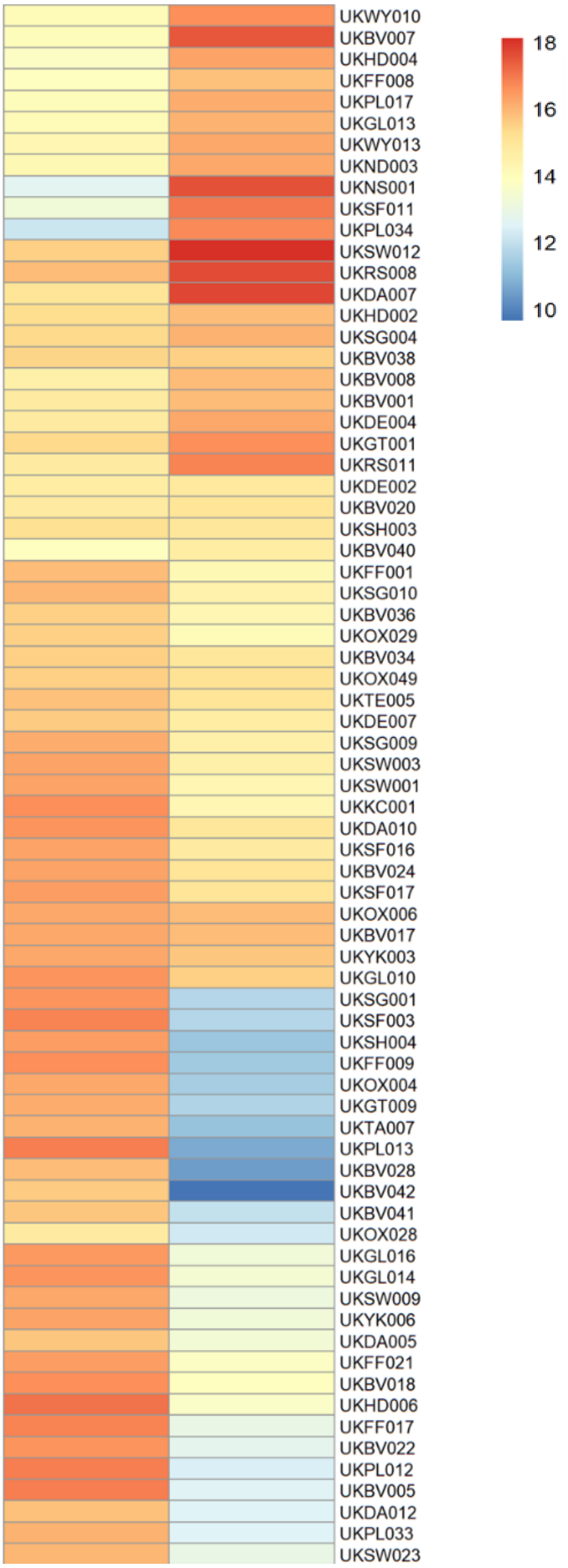

Supplementary Figure S7. Heatmap for the protein levels of Plasminogen and Neutrophil Elastase. The protein levels exhibited a negative correlation. Each row is a sample, and the colour of each cell represents the protein abundance level.

References

1. Demichev V, Messner CB, Vernardis SI, Lilley KS, Ralser M. DIA-NN: neural networks and interference correction enable deep proteome coverage in high throughput. *Nat Methods* 2020: 17(1): 41-44.
2. Kanellakis NI, Wrightson JM, Gerry S, Ilott N, Corcoran JP, Bedawi EO, Asciak R, Nezhentsev A, Sundaralingam A, Hallifax RJ, Economides GM, Bland LR, Daly E, Yao X, Maskell NA, Miller RF, Crook DW, Hinks TSC, Dong T, Psallidas I, Rahman NM. The bacteriology of pleural infection (TORPIDS): an exploratory metagenomics analysis through next generation sequencing. *Lancet Microbe* 2022: 3(4): e294-e302.
3. Corcoran JP, Psallidas I, Gerry S, Piccolo F, Koegelenberg CF, Saba T, Daneshvar C, Fairbairn I, Heinink R, West A, Stanton AE, Holme J, Kastelik JA, Steer H, Downer NJ, Haris M, Baker EH, Everett CF, Pepperell J, Bewick T, Yarmus L, Maldonado F, Khan B, Hart-Thomas A, Hands G, Warwick G, De Fonseka D, Hassan M, Munavvar M, Guhan A, Shahidi M, Pogson Z, Dowson L, Popowicz ND, Saba J, Ward NR, Hallifax RJ, Dobson M, Shaw R, Hedley EL, Sabia A, Robinson B, Collins GS, Davies HE, Yu LM, Miller RF, Maskell NA, Rahman NM. Prospective validation of the RAPID clinical risk prediction score in adult patients with pleural infection: the PILOT study. *Eur Respir J* 2020: 56(5).
4. Rahman NM, Kahan BC, Miller RF, Gleeson FV, Nunn AJ, Maskell NA. A clinical score (RAPID) to identify those at risk for poor outcome at presentation in patients with pleural infection. *Chest* 2014: 145(4): 848-855.
